# Scalable and accessible personalized photodynamic therapy optimization with FullMonte and PDT-SPACE

**DOI:** 10.1117/1.JBO.27.8.083006

**Published:** 2022-04-04

**Authors:** Shuran Wang, Xiao Ying Dai, Shengxiang Ji, Tina Saeidi, Fynn Schwiegelshohn, Abdul-Amir Yassine, Lothar Lilge, Vaughn Betz

**Affiliations:** a University of Toronto, Edward S. Rogers Sr. Department of Electrical and Computer Engineering, Toronto, Ontario, Canada; b University of Toronto, Department of Medical Biophysics, Toronto, Ontario, Canada; c University Health Network, Princess Margaret Cancer Centre, Toronto, Ontario, Canada

**Keywords:** open-source, biophotonics, Monte-Carlo, optimization, photodynamic therapy

## Abstract

**Significance:**

Open-source software packages have been extensively used in the past three decades in medical imaging and diagnostics, aiming to study the feasibility of the application *ex vivo*. Unfortunately, most of the existing open-source tools require some software engineering background to install the prerequisite libraries, choose a suitable computational platform, and combine several software tools to address different applications.

**Aim:**

To facilitate the use of open-source software in medical applications, enabling computational studies of treatment outcomes prior to the complex *in-vivo* setting.

**Approach:**

FullMonteWeb, an open-source, user-friendly web-based software with a graphical user interface for interstitial photodynamic therapy (iPDT) modeling, visualization, and optimization, is introduced. The software can perform Monte Carlo simulations of light propagation in biological tissues, along with iPDT plan optimization. FullMonteWeb installs and runs the required software and libraries on Amazon Web Services (AWS), allowing scalable computing without complex set up.

**Results:**

FullMonteWeb allows simulation of large and small problems on the most appropriate compute hardware, enabling cost improvements of 10× versus always running on a single platform. Case studies in optical property estimation and diffuser placement optimization highlight FullMonteWeb’s versatility.

**Conclusion:**

The FullMonte open source suite enables easier and more cost-effective *in-silico* studies for iPDT.

## 1 Introduction

Visible and near-infrared light (400 to ∼1000  nm) has seen wide adoption in medical applications in recent years^1^^–^^3^ due to its ability to penetrate tissues, its low cost to produce, and the minimal risk of unintended tissue damage at irradiances below $250\;\mathrm{mW}\;{\mathrm{cm}}^{-2}$. These applications range from imaging techniques and diagnostics such as diffuse optical tomography (DOT),^1^ to observing the progression of cancerous tissues in bioluminescence imaging (BLI)^2^ and to treating tumors with light-activated therapies such as photodynamic therapy (PDT)^3^ and photothermal therapy.^4^ With the increase in the number of applications, the need for fast and flexible software tools that can predict and optimize outcomes based on the optical energy delivered has increased. To this end, several open-source software packages that model the light distribution in preclinical and clinical applications have seen the light in the past three decades.^5^^–^^9^

Open-source software, applied in a wide range of engineering and scientific fields including the medical field, generally provide a flexible and fast way to understand the feasibility of realizing a problem under study, decrease the development time needed to repeat work done in the field to improve on it, and encourage widespread use of different tools among the research community. This fact holds true for biomedical translation in particular, where improvement cycles and iterations are not only expensive but also often result in the abandonment of projects. However, open-source software commonly has undesirable traits that can hinder users from fully utilizing the available features. First, open-source applications usually incorporate multiple prebuilt libraries along with custom software source code; this can make them challenging to build and set up for users who do not have a software development background. They also often lack a user-friendly interface and are instead controlled by custom data files or scripts or even require software code modifications. The combination of a challenging set up and a developer-focused user interface creates a significant learning curve that is a barrier to adoption in the target medical biophysics research community. Second, the compute requirements of these tools can be significant and vary depending on the problem being considered—for example, some problems require servers with a large amount of memory, whereas others are best solved with graphics processing units (GPUs). Procuring an appropriate group of servers to solve a problem is time consuming and expensive, and there is no guarantee the servers will be the best fit to the next set of problems to be solved. Third, some applications require the combination of multiple software tools, and often these tools do not have compatible inputs and outputs. For example, PDT modeling—the focus of this paper—first requires the ability to accurately model light propagation in biological tissues and second requires the ability to change the treatment parameters to optimize and tailor the outcome for each individual patient. The final results usually then require additional visualization software for full interpretation.

In this work, we address all three of these challenges. We introduce a web-based interface for interstitial photodynamic therapy (iPDT) modeling and optimization that is user-friendly, flexible in supporting different tumor types and locations, and fast to quickly investigate the effect of different parameters (such as tissue optical properties and intrinsic tissue responsivity) on the treatment outcome *in-silico* without the need to deal with technical and logistic difficulties that come with *in-vivo* direct measurements. Once a problem is entered through the web interface, highly optimized software tools (FullMonteSW^5^ and PDT-SPACE^10^) are installed on a user-chosen Amazon Web Server (AWS) instance, automating the server set up and ensuring that users can choose the appropriate type and amount of compute resource for each problem. The analysis (FullMonteSW) and optimization (PDT-SPACE) tools are integrated and combined with in-browser visualization tools, allowing entire analysis flows to be created and run with no software set up on the user’s machine. In addition, the tools and the web interface software are all open source, allowing researchers to extend and build on this infrastructure.

The rest of this paper is organized as follows. First a background on PDT and the different open-source software packages designed for in-browser PDT analysis and optimization is presented in Secs. 2 and 3. The web-based interface along with the different features supported and a brief tutorial on how to use each one are then described in Sec. 4. Sections 4.2–4.4 discuss the integration with AWS to efficiently choose the most suitable platform based on the problem at hand, its volume, and the required spatial resolution. Finally, the use of the web interface is demonstrated in Sec. 5 through two case studies related to ongoing preclinical research. One case illustrates treatment optimization prior to engaging in actual *in-vivo* experiments. The other case demonstrates the generation of look-up tables to permit real-time extraction of tissue optical properties from experimental data based on strategically placed optical sources. In both cases, the experimental execution is optimized, ensuring that the data extracted from these *in-vitro* work are scientifically relevant. Finally, we conclude in Sec. 6.

## 2 Background

### 2.1 Photodynamic Therapy

PDT, or the use of light activated photosensitizing drugs called photosensitizers (PS), is an approved therapy for various indications in oncology,^11^ infectious diseases,^12^ and other conditions.^13^ The interactions of light photons with the PS generates radicals, predominantly reactive oxygen species, resulting in spatially confined tissue destruction. The majority of the currently approved indications for PDT in oncology target organ surfaces, such as the skin,^14^ esophagus,^15^ or bladder,^16^ whereby access is provided by endoscopy and the tissue target depth is limited to 1 to 3 mm depending on the excitation wavelength. In these situations, typically individual, large surface covering light or photon emitters are used and the fluence rate gradient $\left[\mathrm{mW}\;{\mathrm{cm}}^{-2}\;{\mathrm{mm}}^{-1}\right]$ follows a one-dimensional distribution as a function of depth or radius from that emitter. Treatment of larger solid malignancies, such as in the brain, prostate, or pancreas, poses additional challenges related to adequate light delivery throughout the treatment volume. For interstitial PDT (iPDT), the clinical target volume often presents a complex three-dimensional (3D) shape and size, requiring placement of multiple sources with overlapping fluence rate distributions. To date, source placement and optical power allocation follow mostly empirical treatment plans such as the implementation of iPDT for malignancies in the central nervous system by Stummer et al.^17^^,^^18^

#### 2.1.1 iPDT planning challenges

Present clinical implementation of iPDT planning currently aims to achieve an empirically determined minimal required fluence $\left[J\;{\mathrm{cm}}^{-2}\right]$ at the target boundary commonly based on predetermined light emitter spacing. For some oncological indications, such as the prostate,^19^ iPDT treatment monitoring can be used to substitute partially for treatment planning for a given empirically determined light source placement. Fluorescence and diffuse reflectance measurements representing the localized PS concentration and tissue optical properties are also taken into consideration to determine the duration of optical power delivery at each location.^20^^–^^22^

Increasing iPDT efficacy requires replacing empirical photon source placements by an individualized treatment planning process that considers also spatial emission properties for various optical fiber emitters. Through this individualization of the emitter placement, PDT can enter the age of personalized medicine. In addition, the planning process must be based on a quantitative model, linking PDT efficacy determining parameters – $3O_{2}$, the PS concentration, and the local fluence rate $\left[\mathrm{mW}\;{\mathrm{cm}}^{-2}\right]$ – with a desired outcome. In oncology, the endpoint is typically tissue necrosis; however, planning for apoptotic cell death should be possible.^23^

Optimizing PDT delivery requires evaluating treatment plans based on the ability to destroy 98% to 100% of the target volume while preserving the surrounding tissue, particularly those performing critical functions, as in the brain or the cardiovascular system. Secondary optimization objectives include minimizing the number of invasive light sources to be placed into the malignancy to reduce treatment complexity and minimizing the overall treatment time. As these optimization goals are not necessarily compatible to generate the best clinically implementable plan, the physician is often presented with multiple possible plans—with trade-offs between the aforementioned conflicting objectives—to select from based on the desired outcome. The output for each of the possible plans is the volume fraction of the tumor receiving the minimum required photon dose, the volume of critical normal tissue at risk, as well as the number and emission characteristics of the photon sources, their power and maximum light irradiation times. Hence, the planning process needs to combine quantitative photon distribution simulations with parameter optimization.

### 2.2 Prior Web- and Cloud-Based Monte Carlo Tools

Some web or cloud-based Monte Carlo (MC) simulators for biomedical applications have been published. CloudMc^6^ is a framework that allows an MC program to be launched on the Microsoft Azure cloud, with a portion of the photon packets executed on several different compute nodes, and final statistical results aggregated across nodes. This infrastructure is not applicable to our problem as it supports only Windows-based executables, and our simulation and optimization tools, like most open source projects, are instead developed on Linux. Wang et al.^7^ modify the ESG5 high energy electron and photon MC simulator to run a subset of the photons on each node in an AWS cluster; the lower-energy photons in PDT cannot be accurately simulated in this system. The most comparable work to ours is the MCX Cloud tool suite^24^ which (such as our tools) simulates photon transport in biological tissues. Unlike MCX cloud, we use a public (AWS) cloud instead of a private cluster, and our tool suite includes not only Monte Carlo simulation of photon transport but also optimization of iPDT. Multiscattering^25^^,^^26^ is a GPU-accelerated MC software with a web interface supporting quick and easy setup for users. It uses voxels to represent the problem geometry, rather than the tetrahedral mesh representation used by FullMonte and MMC, and has additional features for simulating spherical particles. Simulations are run on a cluster of three computers at Lund University, each of which has four GPUs.

## 3 Open-Source Tool Suite for PDT

**Fig. 1:**
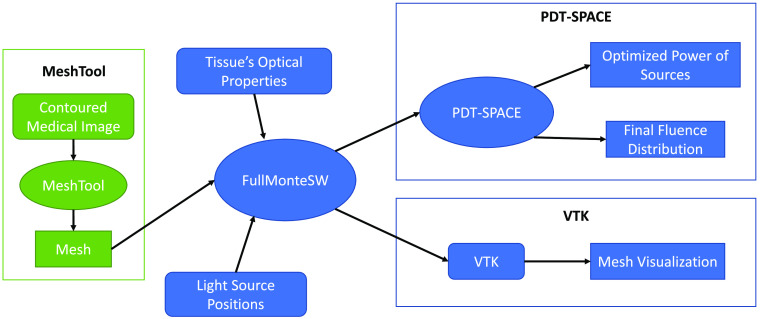
Process flow of the iPDT software tools.

Figure 1 shows how the data preparation, photon simulation, and PDT optimization tools in our open source suite interact. The geometry of a case of interest must first be represented as a tetrahedral mesh; our MeshTool facilitates creating this mesh from contoured medical imaging data. This mesh along with tissue properties and light source information are used by FullMonteSW to simulate light propagation, and PDT-SPACE can optimize a treatment plan by simulating various options with FullMonteSW.

### 3.1 Photon Simulation: FullMonteSW

**Fig. 2:**
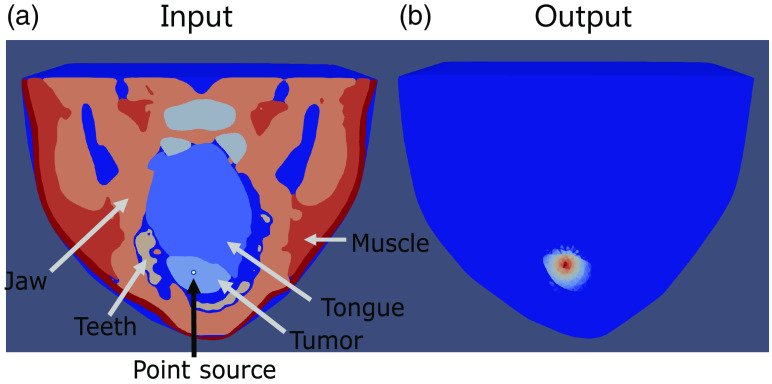
Example of the paraview visualization of an oral tumor at the base of the tongue with (a) a single point source inserted into the tongue and (b) the output fluence visualization from a light irradiation modeled for 633 nm.

FullMonteSW^5^ performs Monte Carlo simulations of light propagation within 3D biomaterials of arbitrary shapes and tissue types to evaluate PDT treatment plans as well as for other light propagation studies in clinical^16^ and preclinical research.^27^^,^^28^ The tool simulates the light propagation by tracing a user-specified number of photon packets^29^ and aggregating statistics on photon absorption throughout the volume. It supports a variety of light sources, including point sources, radially emitting fibers, cut-end fibers emitting in a cone, arbitrary surface emitters, and combinations of these. Geometrically, it can model arbitrary 3D volumes with a tetrahedral mesh structure, allowing for accurate modeling of smooth and irregular surfaces, which are common in biological tissues. Finally, FullMonteSW is able to collect several user-defined output values, including the energy absorbed per tetrahedral element, and the energy entering or exiting internal or external surfaces. As shown in Fig. 1, FullMonteSW takes as input the optical properties of each tissue region, a 3D tetrahedral mesh that indicates each tissue region, the light source positions, their emission profiles, and their powers. The output data are used to either aid in iPDT optimization using the PDT-SPACE tool or directly visualize the fluence and the mesh structure using open-source tools such as Paraview.^30^ Figure 2 shows an example of the visualization of an input and output mesh of an oral tumor at the base of a tongue with a single optical source.

FullMonteSW employs the Henyey–Greenstein scattering phase function, as the deep tissue tumor cases targeted employ lambertien or isotropic light sources. Since the code is open source, other scattering phase functions^31^^–^^33^ can be added by end users if required for other applications. As described in Ref. 5, FullMonteSW has been cross-validated against two other tetrahedral Monte Carlo photon simulators (TimOS^34^ and MMC^35^) on 3D geometries, as well as MCML^36^ on simpler layered geometries. FullMonteSW was also validated against analytic solutions using diffusion theory for two cases: an isotropic point source in a homogeneous infinite medium and a pencil beam in a homogeneous semi-infinite medium. FullMonteSW has internal consistency checks that are always active to ensure that physical laws such as conservation of energy are respected by the simulation outputs. FullMonte has a large regression suite comprising both system tests that check the final result accuracy and unit tests that validate internal software components. These tests are run and summarized automatically on every change to the software code; while the tests consume over 36 h of cpu time, they are run in parallel so they complete in ∼1.5  h.

Apart from PDT, FullMonteSW can be used to simulate light propagation for other applications, such as disinfection of N95 filter facepiece respirators (FFR) using ultraviolet light germicidal irradiation,^37^^–^^39^ and predicting the observed light distribution in imaging applications such as BLI imaging and trans-illumination spectroscopy for breast cancer detection.^40^

#### 3.1.1 FullMonteSW runtime

FullMonteSW’s runtime is primarily affected by the number of photon packets to simulate. A higher packet count results in a higher signal-to-noise ratio. The value varies widely and depends on the indication and the desired spatial resolution; however, $10^{6}$ to $10^{7}$ photon packets are usually sufficient. For studies requiring submillimeter resolution, as in the case of UVC inactivation of filter FFRs^37^^,^^38^ and thin light absorbing layers in the skin or the eye’s pigment epithelium, up to $10^{9}$ photon packets may be needed. This makes high computational performance a priority. FullMonteSW uses both single-instruction multiple data instructions and multithreading (to use multiple cores) and is currently the fastest software 3D tetrahedral Monte Carlo simulator.^5^ If further performance is required, both GPU (FullMonteCUDA)^41^ and FPGA (FullMonteFPGACL)^42^ accelerated versions are available. These accelerated versions can run 5× to 10× faster than FullMonteSW for many (but not all) problems, and they are among the highest performance accelerated tetrahedral Monte Carlo simulators.

### 3.2 PDT Optimization: PDT-SPACE

**Fig. 3:**
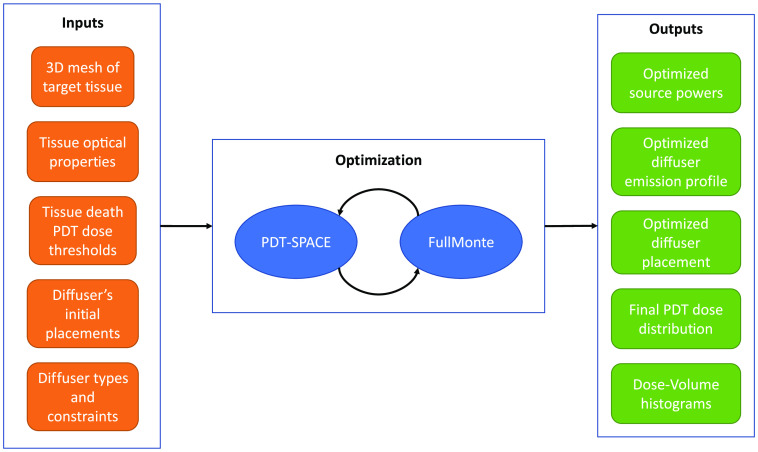
PDT-SPACE overall flow.

The PDT-SPACE tool^10^ automates the treatment planning process by optimizing several degrees of freedom to achieve a desired tumor coverage while minimizing damage to the surrounding healthy organs at risk (OARs); its inputs and outputs are shown in Fig. 3. Given a set of light source locations and types, PDT-SPACE uses a convex optimization formulation to determine the power input to each source^43^ to simultaneously maintain the tumor volume destroyed well above 98% while minimizing the OAR damage. In addition, PDT-SPACE can optimize the emission profile of cylindrical diffusers under the same goal while respecting user-specified manufacturability constraints.^10^ PDT-SPACE can also optimize the positions of the light sources to further improve the quality of the treatment plan. It treats the light source positions specified by the clinician as starting conditions and then uses a combination of simulated annealing and reinforcement learning to iteratively propose and evaluate improved probe positions.^44^

In each of these usage modes, PDT-SPACE must repeatedly call FullMonteSW to determine the light distribution throughout the tissue for the proposed plans. The program uses the result to evaluate plan quality against predetermined outcome conditions. Usually, $10^{6}$ photon packets are simulated in FullMonteSW to achieve sufficiently accurate results to guide optimization. PDT-SPACE also employs efficient search techniques to minimize the number of FullMonteSW calls and effectively the overall runtime.

### 3.3 Open-Source Code and Docker Containers

FullMonteSW and PDT-SPACE are open-source and can be found on Gitlab repositories. Links to the repositories can be found on Ref. 45, and the wiki pages within the repositories provide extensive documentation on how to use and script the tools. These repositories also have extensive regression tests that are automatically launched whenever a source code change is committed to the repository, making it easier and safer for developers to update the code.

Both FullMonteSW and PDT-SPACE are complex programs that have dependencies on several software libraries and packages. Installing these dependencies can be daunting on some operating systems, so both tools support the alternative option of installing and running within a prebuilt docker container. Docker containers provide portable encapsulation of an environment to be run under any operating system, greatly reducing the time and effort required to get all the dependent software installed. The Wiki pages include instructions on docker installation and execution of the tools; the docker images are rebuilt and updated automatically whenever a source code change is committed so they are always up to date.

## 4 FullMonteWeb

To make it easier for researchers to use the FullMonteSW and PDT-SPACE tools, we have developed *FullMonteWeb*. FullMonteWeb allows entry of a wide range of biophotonic and PDT-related problem definitions through web forms, launch of the computation on a user-chosen Amazon Web Services (AWS) instance, and visualization and downloading of results from within the same web browser interface. All the FullMonte tools, including FullMonteWeb, are available from Ref. 45. FullMonteWeb is written in Python and leverages the django framework.^46^

### 4.1 Flow and Input Data

**Fig. 4:**
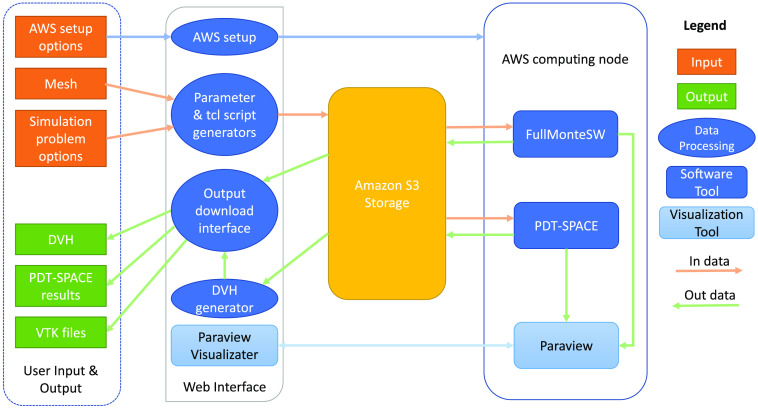
FullMonteWeb overall flow.

**Fig. 5:**
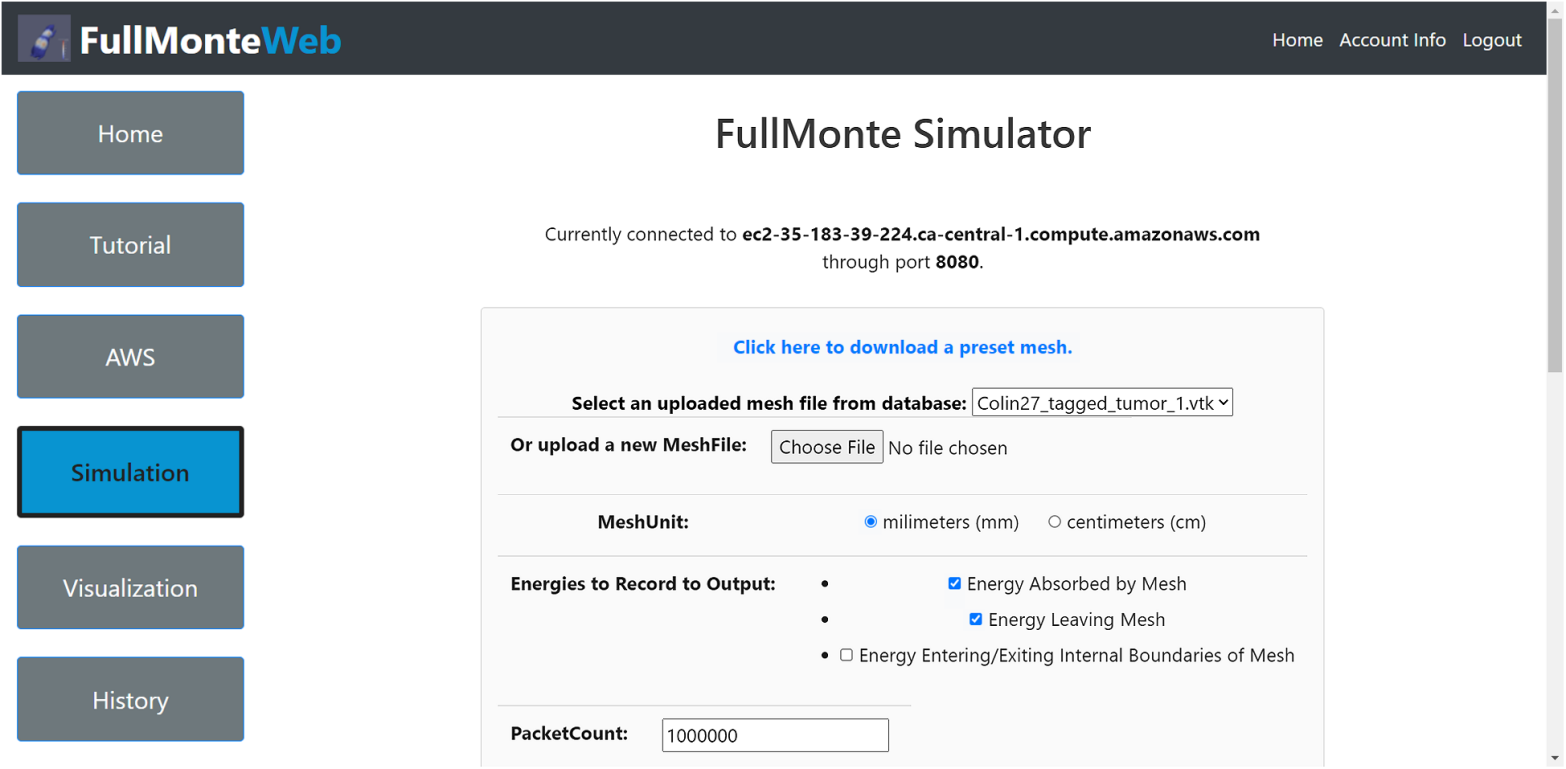
FullMonte simulation main page.

Figure 4 shows the usage and architecture of FullMonteWeb. User input is shown in orange, output is green, the open-source FullMonte tools are dark blue, whereas other open-source tools we leverage are shown in light blue. Users can upload a.vtk^47^ format mesh that details the geometry of the tissues of interest or choose from a set of preloaded meshes. If an user is starting from medical imaging data, they can use tools such as ITK-snap^48^ to segment the images then use the MeshTool available on the FullMonte website to create a tetrahedral mesh, which can be uploaded as input to FullMonteWeb. The FullMonteWeb web forms guide the user to enter the other inputs defining a problem (e.g., light sources, material optical properties, packet count to simulate, and desired outputs to compute). These forms validate that the entered data is self-consistent and physically possible and link to embedded tutorials that give examples of how to set up complete problems. Figure 5 shows a screenshot of one of the web forms used to set up a simulation; please see the Appendix for a more extensive walkthrough.

Entering the parameters defining a simulation in web forms is user-friendly, but it can limit the range of simulations possible unless a very large (possibly overwhelming) number of options are exposed in the forms. An alternative to option entry is scripting: the core solver, input and output routines in FullMonteSW can be directly called from scripts written in either the python or Tcl languages. This gives tremendous flexibility in problem set up by writing a new script that invokes the various input, output, and solver routines in different orders or even in loops. However, it can be a more difficult flow to learn as it requires some programming experience. To allow efficient use by both novice and power users, FullMonteWeb takes a hybrid approach. The most common options to FullMonteSW and PDT-SPACE are presented in web forms. Once all the forms have been completed, a Tcl script that implements the desired simulation or optimization flow is created and displayed as shown in Fig. 4. The script can be used as-is or the user can edit it in any way he or she desires. Editing the script allows specification of less common options, custom simulation flows, or loops that automate sweeping of parameters such as optical properties or other repetitive simulations required for statistical analysis. Once the Tcl script is finalized, it is uploaded to Amazon’s S3 storage and used to drive the simulation run on the AWS instance. Table 1 lists some of the options available directly in web forms, along with some of the power-user options and flows that Tcl scripts enable.

**Table 1:** Available options for simulation.

	Web form options	Additional scripting options
FullMonte simulation	Mesh file (VTK format)	Specify mesh in other supported formats (Comsol, TimOS, text)
	Light sources (any of point, pencil beam, volume, ball, cylinder, surface)	Specify other supported source types (tetra face)
	Outputs to compute (fluence throughout volume, fluence across surface, absorbed power)	Custom outputs
	Unit specification	Run kernel multiple times with different parameters (energy and number of packets)
	Material optical properties	Track time/memory at any point
	—	Arbitrary control flow/custom simulation
PDT-SPACE simulation	Mesh file	Scripting not supported for PDT-SPACE simulation
	Optical properties	
	Dose thresholds	
	Probe types	
	Initial source placement	

**Fig. 6:**
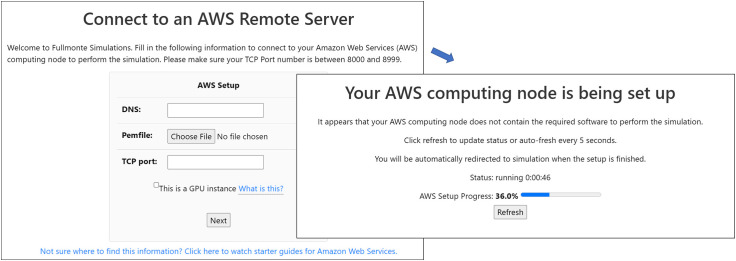
AWS setup feature to allow scalable computing.

### 4.2 AWS Integration

To leverage the power of cloud computing services, a user is required to launch an AWS Elastic Compute Cloud (EC2) instance (a virtual machine that allows access to some or all of the resources in a particular computer server) and provide its DNS name and.pem permission file to FullMonteWeb, embedded tutorials again guide the user through this operation. As shown in Fig. 6, FullMonteWeb automatically sets up this AWS instance. It performs a remote ssh (Secure Shell) operation to connect to the instance and scans the instance for the required environment and software (Docker, FullMonteSW, Paraview Visualizer, PDT-SPACE, as well as FullMonteCUDA^41^ and NVIDIA GPU drivers if the instance contains a GPU). FullMonteWeb will install only missing components on the user’s behalf, allowing simple operation but minimizing setup time if repeated simulations are run. Users can scale compute power not only by choosing a more powerful AWS instance but also by having multiple long-running simulations proceed in parallel by specifying and launching simulations on multiple AWS instances.

**Fig. 7:**
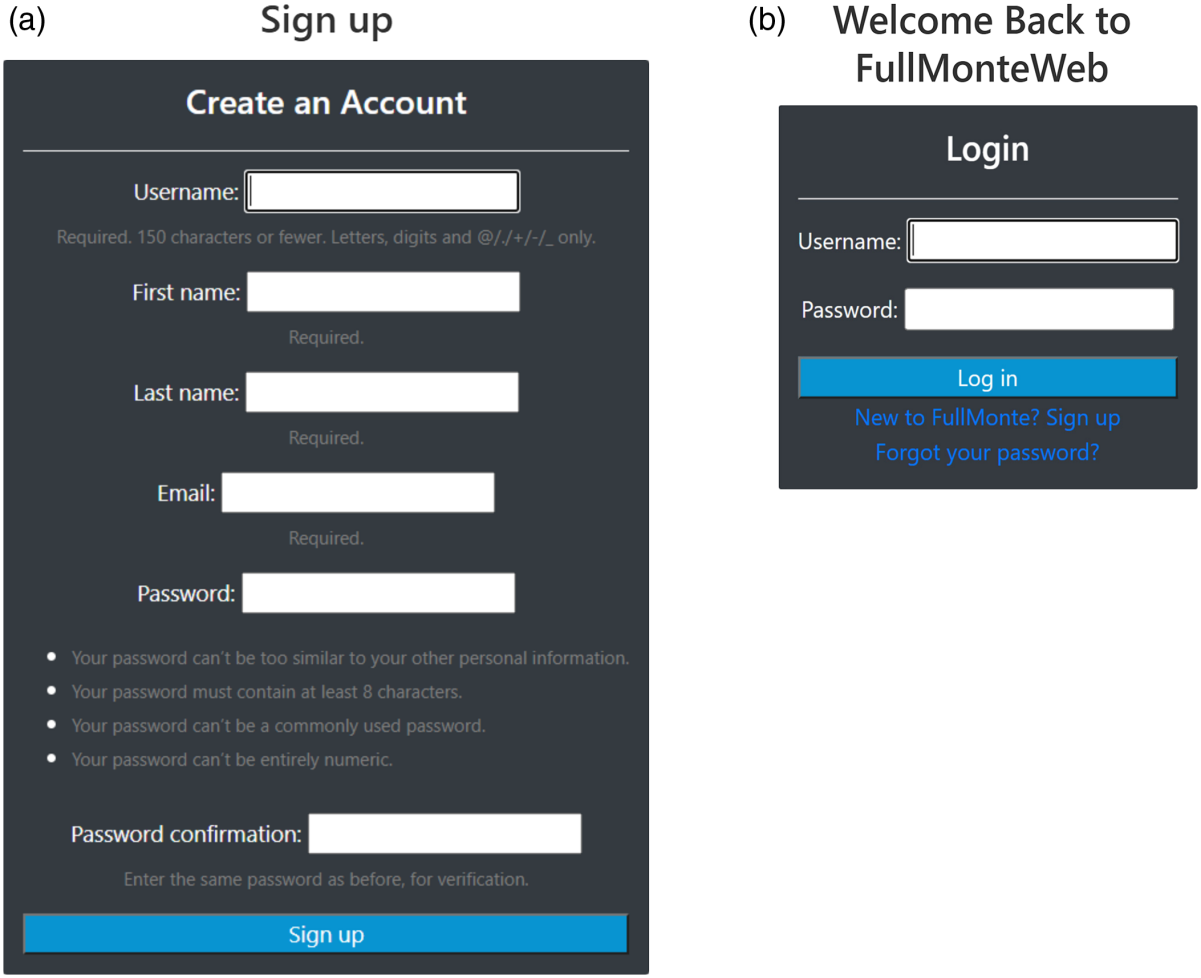
(a) Registration and (b) login pages to allow per-user compute and simulation data.

**Fig. 8:**
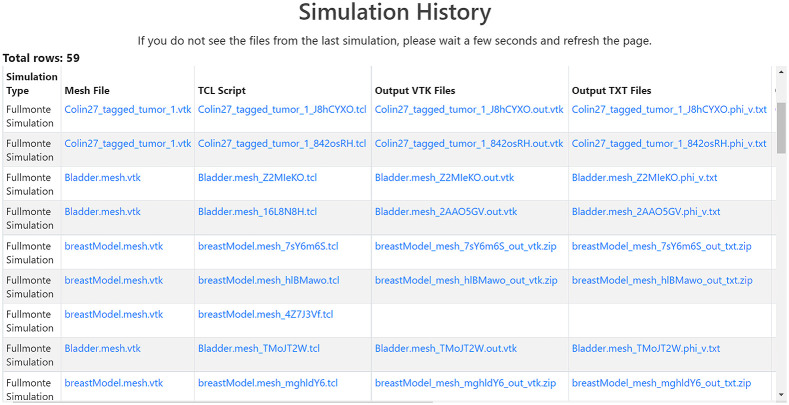
History files download feature.

To ensure user data and compute resources are kept private, FullMonteWeb keeps a separate account for each user as shown in Fig. 7; sign-up is free. Recent simulation inputs and outputs are stored in the Amazon S3 storage cloud as shown in Fig. 4 and can be accessed through a history view in FullMonteWeb, as shown in Fig. 8.

### 4.3 Integrated Visualization

The outputs of a simulation are indicated in green in Fig. 4. They include report files detailing the dose-volume histogram (DVH) of a treatment plan for both the target tissue and the organs-at-risk; if plan optimization was performed with PDT-SPACE, the plan itself (probe locations, types and powers) is reported. Outputs such as the light fluence at each point in the 3D treatment volume are written into a vtk-format mesh file along with the mesh geometry itself so it can be visualized and processed with Paraview^30^ or similar tools. FullMonteWeb also includes two integrated visualization tools so the key result data can be directly examined graphically in the web browser, without any installation of tools on the user’s computer.

#### 4.3.1 Interactive dose-volume histograms

FullMonteWeb generates an interactive DVH by building on the matplotlib and mpld3 python graphing libraries. The user can select which tissue regions to display on the DVH, examine individual data values by hovering the mouse over them, and zoom in and out on portions of the plot. The DVH data are retained in the Amazon S3 storage for independent access or downloading to the user’s computer.

#### 4.3.2 Interactive 3D visualization

**Fig. 9:**
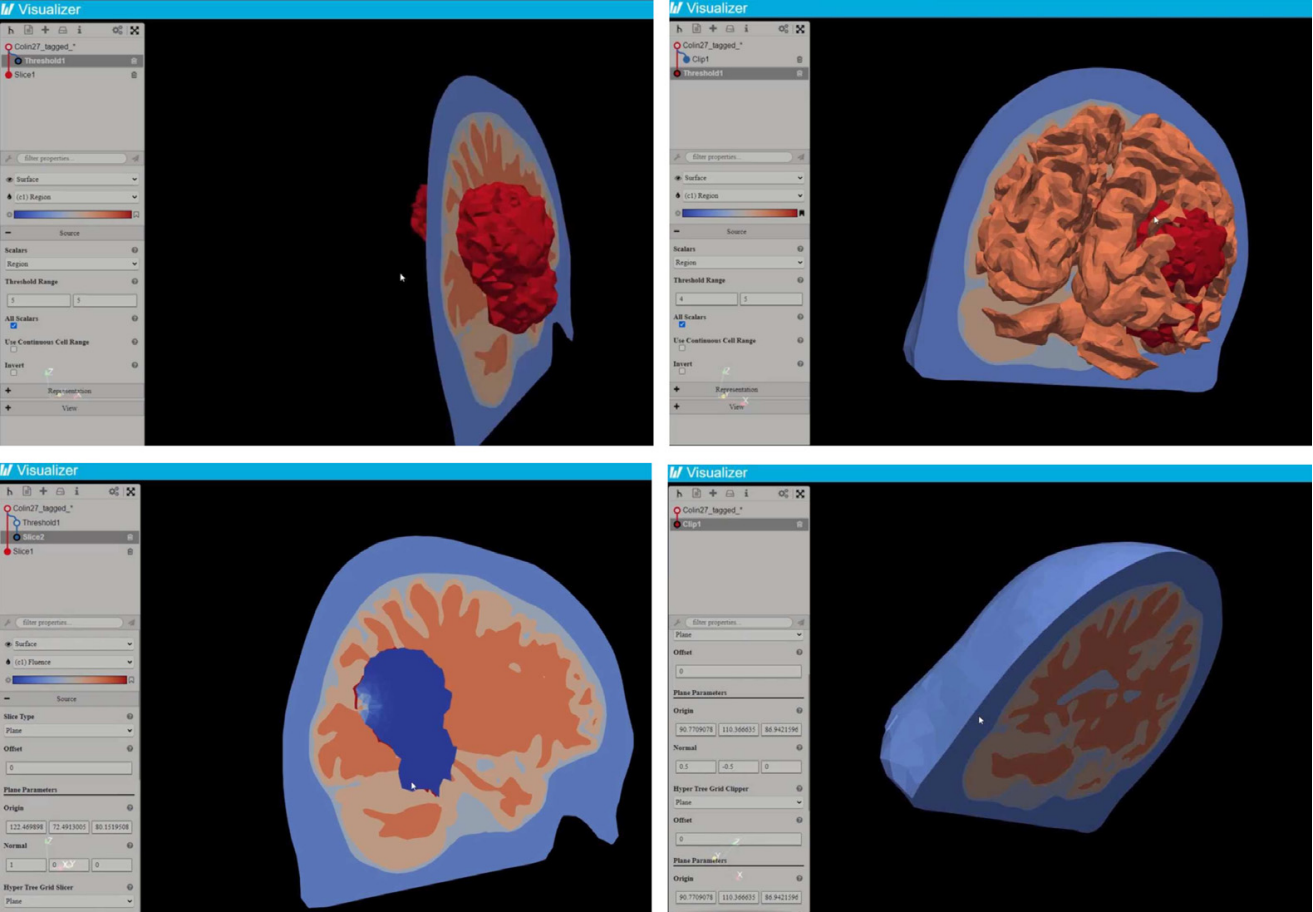
3D visualizer example views on a brain mesh and the resulting fluence.

The 3D visualization feature allows users to visualize the mesh and resulting fluence in 3D without the need for state-of-the-art graphics cards or specialized software on the user’s device. FullMonteWeb leverages the third party 3D visualization browser-based ParaView Visualizer software.^49^ As shown in Fig. 4, the full Paraview application is automatically installed and run on the EC2 instance by FullMonteWeb. Paraview renders the 3D geometry and important outputs such as fluence in response to interactive user input, and the rendered data are sent to the Paraview Visualizer running on the user’s web browser for display. This arrangement keeps the more complex and computationally demanding software on the EC2 instance, with only the final display being performed in the user’s browser. The mesh and various tissue regions can be visualized before launching a simulation simply by clicking the “Open 3D Interactive Visualizer” button in FullMonteWeb; this can help in choosing the desired initial light source positions for a given case. After simulation completes, the resulting fluence can be visualized along with the tissue geometry. Figure 9 shows a few of the views that can be generated from a treatment plan output. The visualizer can also be used to review prior results stored in the S3 cloud or uploaded (in vtk format) by the user without rerunning the simulator.

### 4.4 Efficiency versus EC2 Instance

One of the goals of FullMonteWeb is to abstract and configure compute resources so that researchers can choose the appropriate type and amount of CPUs, GPUs, and memory for any particular PDT simulation or optimization. There are usually two main metrics of concern to researchers: time to result and cost (while maintaining accuracy). We explore how the chosen EC2 instance affects these two metrics for simulations that vary in mesh size and whether a treatment plan is being evaluated with FullMonteSW or a plan is being created by the combination of PDT-SPACE and FullMonteSW.

Table 2 summarizes the six different EC2 instances we test. Each EC2 instance is a virtual machine with access to a certain number of virtual CPUs (vCPUs) and physical RAM. The Intel and AMD processors tested can run two threads on a single compute core (using a technology called hyperthreading or simultaneous multithreading) so Amazon counts a single core as two vCPUs. The instances we compare range in size from one vCPU to 64 vCPUs and have between 0.5 and 256 GB of physical RAM; two of the instances also include Nvidia GPUs. As Table 2 shows, the price per hour of an EC2 instance increases rapidly as its memory size and compute capabilities increase and the platforms we compare vary by over 500× in cost per hour.

**Table 2:** EC2 instance details.

Name	vCPUs	CPU type	GPUs	GPU type	CPU RAM	GPU RAM	Price ($USD/h)
t2.nano	1	Intel(R) Xeon(R) CPU E5-2676 v3 @ 2.40 GHz	0	N/A	0.5 GB	N/A	0.0058
t3a.xlarge	4	AMD EPYC 7571	0	N/A	16 GB	N/A	0.1504
t3a.2xlarge	8	AMD EPYC 7571	0	N/A	32 GB	N/A	0.3008
m6i.16xlarge	64	Intel(R) Xeon(R) Platinum 8375C CPU @ 2.90 GHz	0	N/A	256 GB	N/A	3.072
g4dn.xlarge	4	Intel(R) Xeon(R) Platinum 8259CL CPU @ 2.50 GHz	1	Nvidia Tesla T4	16 GB	16 GB	0.526
p3.2xlarge	8	Intel(R) Xeon(R) CPU E5-2686 v4 @ 2.30 GHz	1	Nvidia Tesla V100-SXM2-16 GB	61 GB	16 GB	3.06

**Fig. 10:**
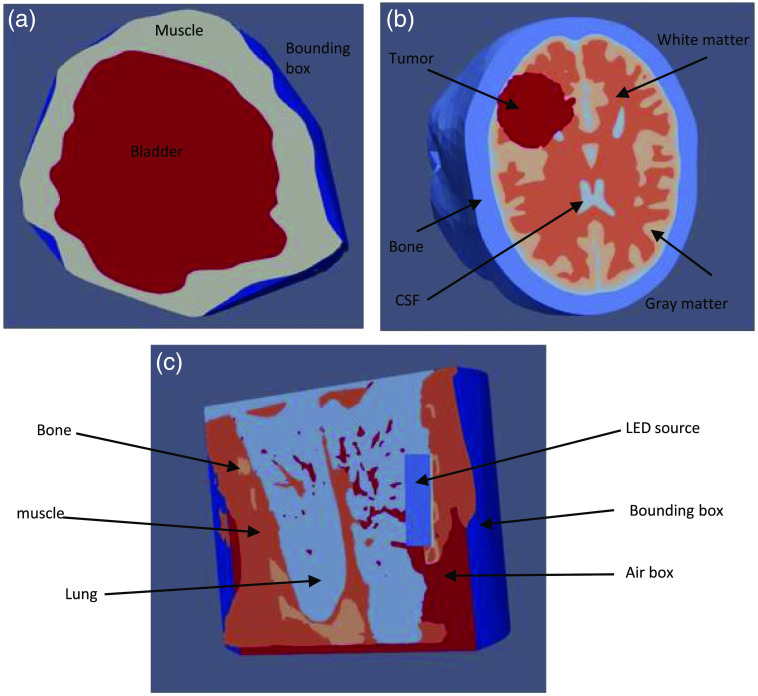
The three models used in measuring the cost-runtime trade-off of the different EC2 instances. (a) A bladder mesh, (a) a low-solution human brain mesh (Colin27), and (c) a pig lung mesh.

Table 3 details how the various EC2 instances perform on plan evaluation tasks using FullMonteSW simulations. For plan evaluation, we simulate three different meshes: a bladder mesh^16^ with a single point light source, the Colin27 brain atlas^35^ (a low-resolution human brain mesh) with 1 point light source, and a high-resolution pig lung mesh—taken from Ramadan et al.^28^—with light emitted from a complex surface source. Figure 10 shows a visualization of the three meshes. All simulations use $10^{7}$ photon packets, a typical value for accurate plan evaluation. The run times in Table 3 include all processing in FullMonteSW or FullMonteCUDA: loading of the mesh into memory, Monte Carlo simulation of $10^{7}$ photon packets, and generating output reports and an output vtk mesh for visualization. The time to set up the docker environment on the EC2 instance is 6 to 8 min but only occurs once and then is cached and reused, so it is not shown in Table 3. The time to upload an input mesh varies between 5 s (for the bladder case) and 1 min (for the pig lung case); once uploaded it is kept in persistent cloud storage so this is required only once per mesh, and hence it is not included in Table 3.

**Table 3:** Compute time and cost for a $10^{7}$ photon packet simulation of a (i) bladder mesh, (ii) low-resolution human brain mesh, and (iii) pig lung mesh. See Fig. 10 for a visualization of the three meshes.

Mesh	Disk usage (MB)	Number of tetrahedra	Instance type	# of vCPU	# of GPU	Memory consumption	FullMonte run time (hh:mm:ss)	Price ($USD/100 runs)
Bladder	8.7	301,079	t2.nano	1	0	224 MB	00:10:50	0.10
			t3a.xlarge	4	0		00:03:21	0.84
			t3a.2xlarge	8	0		00:01:41	0.84
			m6i.16xlarge	64	0		00:00:15	1.28
			g4dn.xlarge	4	1	763 MB	00:00:11	0.16
			p3.2xlarge	8	1	755 Mb	00:00:11	0.94
Human brain	15	423,377	t2.nano	1	0	309 MB	08:51:29	5.13
			t3a.xlarge	4	0		00:23:30	5.89
			t3a.2xlarge	8	0		00:11:27	5.74
			m6i.16xlarge	64	0		00:01:32	7.85
			g4dn.xlarge	4	1	819 MB	00:00:24	0.35
			p3.2xlarge	8	1	811 MB	00:00:16	1.36
Pig lung	1310	45,913,408	t2.nano	1	0	N/A	N/A	N/A
			t3a.xlarge	4	0			
			t3a.2xlarge	8	0	28.1 GB	00:17:12	8.62
			m6i.16xlarge	64	0		00:08:05	41.39
			g4dn.xlarge	4	1	N/A	N/A	N/A
			p3.2xlarge	8	1	28.1 GB	00:10:44	64.4

The FullMonte runtime varies significantly across the meshes. The bladder case executes quickly because the photons have few interactions as they cross the transparent bladder void. The pig lung mesh executes slowly as photons frequently cross tetrahedral boundaries, requiring lookup of optical properties in the entered tetrahedron, and frequently cross tissue regions, requiring additional reflection and refraction calculations. Table 4 shows the relative performance of PDT plan optimization via PDT-SPACE using the Colin27 brain atlas and two different tumor cases, one small ($32\;{\mathrm{cm}}^{3}$ and three light sources) and one large ($104\;{\mathrm{cm}}^{3}$ and 19 light sources), which are cases T7 and T2 from Yassine et al.,^50^ respectively. Note that PDT-SPACE currently supports only CPU-based implementations.

**Table 4:** Compute time and cost for PDT-SPACE optimization of two tumor cases, within the low-resolution human brain mesh. $10^{7}$ photon packets are used in the required Monte Carlo simulations.

Case	Disk usage (MB)	# of tumor tetra	# of nontumor tetra	Instance type	# of vCPU	Memory consumption	Run time (mm:ss)	Price ($USD/ 100 runs)
Small brain tumor	14.7	5,889	417,487	t3a.xlarge	4	422 MB	09:12	2.31
				t3a.2xlarge	8		05:12	2.61
				m6i.16xlarge	64		01:15	6.4
Large brain tumor	14.7	11,079	412,297	t3a.xlarge	4	678 MB	53:34	13.43
				t3a.2xlarge	8		29:54	14.99
				m6i.16xlarge	64		05:19	27.22

While the t2.nano EC2 instance (1 vCPU) is the slowest platform, it is also the most cost-effective platform for the FullMonteSW plan evaluations on the smallest (bladder) mesh. However, its low memory leads to poor performance on the (larger) low-resolution human brain mesh, and it cannot run the pig lung mesh at all, showing that larger instances are essential for some cases. GPU-enabled EC2 instances are the fastest platform for the two smaller meshes, with the fastest GPU (Nvidia Tesla V100) platform outperforming the fastest (64 vCPU) CPU platform by nearly 6×. For the bladder and the low-resolution brain mesh, the GPU-based instances are also more cost-effective than instances with a large number of vCPUs as their higher cost/hour is more than compensated for by their high performance, particularly for the less expensive Nvidia Tesla T4 platform.

On the other hand, the largest (pig lung) mesh, which contains 45.9 million tetrahedra, performs best on CPU-based platforms. This case has a high memory requirement of 28.1 GB and will not run on the two smallest CPU instances or the smaller GPU instance. The fastest platform is the 64 vCPU platform, and a high-end GPU platform of comparable cost is 1.3× slower. This case has both a large mesh and a complex (surface) light source, resulting in a larger preprocessing time before the main Monte Carlo photon simulation begins. This time is reduced by a more capable CPU platform, as the preprocessing code does not leverage a GPU. While the GPU instance still outperforms even 64 vCPUs on the core Monte Carlo loop, it is not enough to overcome the slower preprocessing time due to its lower vCPU count. The most cost effective platform for this mesh is the 8 vCPU platform; its runtime is 2.1× longer than the 64 vCPU instance, but due to its lower price per hour it is 4.8× more cost-effective. This is again impacted by the higher preprocessing time in this test case. The mesh loading and preprocessing code is less parallel than the core Monte Carlo photon simulation loop, so the higher time spent in these operations in the pig lung case helps make the 8 vCPU instance the most cost-effective.

Table 4 shows that PDT-SPACE run time scales well as the number of vCPUs increases; on the large brain tumor case, a 64 vCPU instance reduces compute time by 10× versus a 4 vCPU instance. The scaling is not perfectly linear partially because there is some serial code in PDT-SPACE and partially because the different vCPUs can compete for memory access in parallel code. While the largest (64 vCPU) instance is the fastest, the smaller (8 vCPU) is the most cost-effective, reducing the cost of a simulation by over 2× versus the 64 vCPU instance.

The results show that both the fastest and the most cost-effective platform vary significantly with the problem being solved, highlighting the utility of users being able to choose from the over 100 types of EC2 instances to match compute to the problem at hand. Renting EC2 instances on demand also allows users to scale up their compute as needed, whereas buying and managing their own pools of servers is less flexible in adapting to demand.

## 5 Preclinical Case Studies

Translation of the iPDT plan based on optimized light source placement into the clinic requires demonstration of therapeutic efficacy improvements in large animal models. These models often include canines, pigs, and even higher phylogenetic species. Besides the ethical issues in experimenting on phylogenetic high species, these studies are generally cost and time-intensive. Resolving light propagation issues prior to the onset study can address Russell and Burch’s three R’s in preclinical studies: replace, refine, and reduce.^51^ Replacement of animal experimentation can be achieved by calculating the signal of photon sensors placed noninvasively at the tissue necrosis or coagulation boundaries over the anticipated range of tissue optical properties, instead of harvesting tissue samples from multiple animals to measure optical properties. Refinement and reduction can be achieved by verifying light source placement based on clinical imaging and performing photon distribution simulation to ensure that the anticipated biological effect can be measured in the organs of interest. Below we summarize how we leveraged the presented web-based suite to implement the aforementioned two experiments.

### 5.1 Optical Property Estimation

**Fig. 11:**
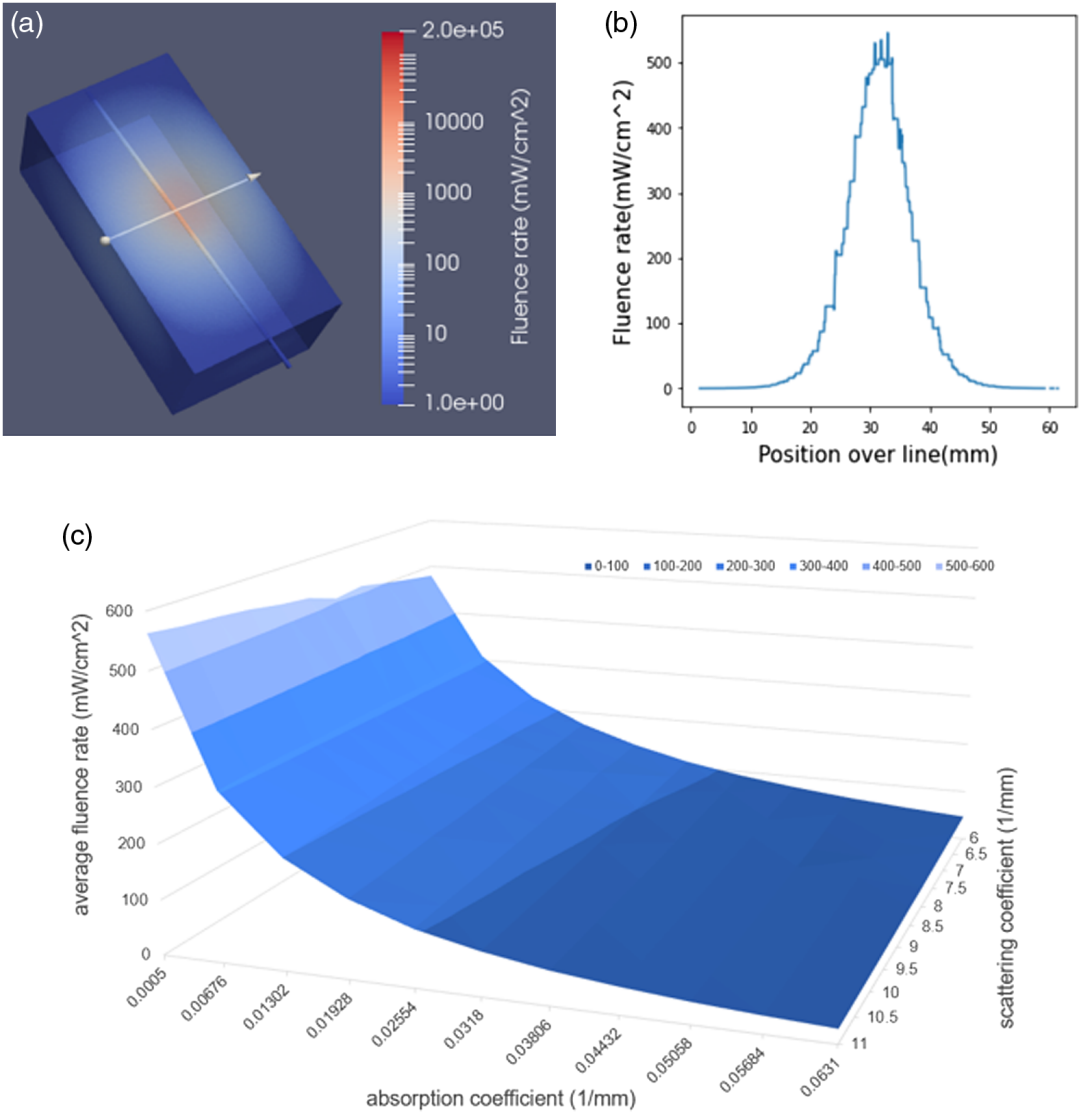
(a) Digital 3D liver phantom used for FullMonteWeb simulation, (b) exemplary dose attenuation curve along the solid white line shown on the phantom, and (c) simulation output showing the predicted fluence-rate at 2 mm from the center line as a function of the absorption and scattering coefficients.

Using 3D CAD tissue phantoms, we leveraged FullMonteWeb to generate tissue optical properties look-up tables that correlate the anticipated light intensity at sensors or the extent of tissue necrosis for planned experiments for a range of diffuser depths inside the liver. Figure 11(a) shows a 6×12×6  cm cube, created using Autodesk Fusion 360 and converted into a.vtk format mesh, representing a liver tissue with a 2-cm long light diffuser placed at an oblique angle into the bile duct. The light diffuser is modeled as a separate region that emits photons diffusely (at a $100\;\mathrm{mW}\;{\mathrm{cm}}^{-1}$). Figure 11(b) indicates the fluence rate $\left[\mathrm{mW}\;{\mathrm{cm}}^{-2}\right]$ over the line shown in Fig. 11(a) for a range of $\mu_{a}$ and $\mu_{s^{\prime }}$ in liver tissue. A total of 11 $\mu_{a}[0.0005-0.0631\;{\mathrm{mm}}^{-1}]$ and 11 $\mu_{s^{\prime }}-[6-11\;{\mathrm{mm}}^{-1}]$ values were assigned to the liver tissue according to published values.^52^^,^^53^ Thus, the simulation run comprises 121 optical properties executed in a single run. To perform this custom simulation, we leveraged the ability to edit the Tcl script generated by FullMonteWeb to add loops over optical characteristics; a simulation is performed for each possible pair of optical characteristics. FullMonteWeb executes the entire simulation, and the user can download a zip file with the output files for all 121 cases. Figure 11(c) shows the FullMonteWeb generated light detected at position r=2  cm on the line shown in Fig. 11(a), as a function of $\mu_{a}$ and $\mu_{s^{\prime }}$. Utilizing multiple detector distances enables determination of the average tissue optical properties in the region of interest during subsequent *in-vivo* experiments in real time. It is important for the user to determine the required number of photon packets to be launched to achieve a sufficient fluence rate signal-to-noise ratio (S/N) for the highest $\mu_{\mathrm{eff}}$ and largest radial distance to be simulated.

Also, the disk storage size requested for these simulations given the number of tissue optical property combinations to be executed must be estimated as all simulation results are kept on disk on the EC2 instance prior to writing back to the Amazon S3 storage. Running a single test model indicated that the sufficient disk storage for one simulation is about 200 MB. Accordingly, a disk storage size of 64 GB was selected, to leave some room for required software installations. Simulated using $10^{7}$ photons on a m6i.16xlarge instance with 64 vCPUs, the overall run time of the 121 cases is 5 h and 11 min. The results can be visualized immediately on the EC2 instance and (if desired) individual result files can also be immediately downloaded to the end user machine. All the results are also compressed and transferred to persistent cloud storage (Amazon S3 storage) so they can be viewed from the FullMonteWeb history page (see Fig. 8) at any later date. For this large simulation, 15 GB of output data is produced, which requires 35 min to compress to a 5-GB.zip file, and a further 55 min to transfer to archival storage.

### 5.2 Diffuser Placement Optimization

**Fig. 12:**
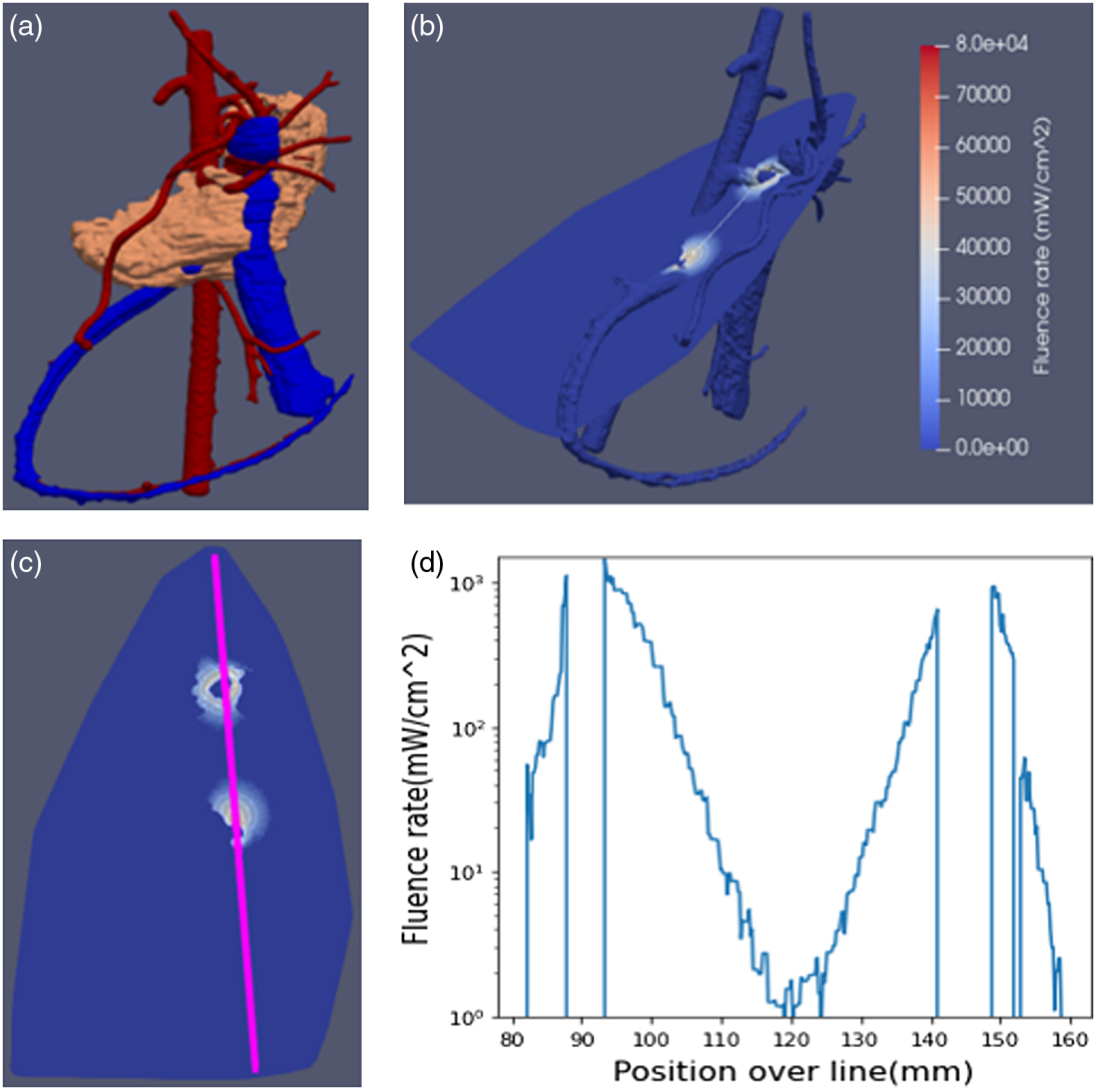
(a) 3D model of pig pancreas (orange) with local arteries (red) and veins (blue), (b) pancreas cross section with color-coded fluence rate for two 10 mm long diffusers embedded in the superior mesenteric artery, (c) visualization of fluence perpendicular to the light emitter, and (d) the fluence rate intensity distribution along the center after establishing the maximum permitted power density to the intima.

An example addressing refinement and reduction in preclinical subject usage pertains to optimizing the placement of optical diffusers within the vascular network in and around the pancreas, whereby the maximum permissible fluence needs to be determined to still permit individual scoring of the tissue response at each optical diffusers by pathology and histology. Here, FullMonteWeb was used to determine the location and power deliverable to all potential vessel locations and power per diffuser as part of a dose-escalation study in a porcine pancreas model. The fluence rates from multiple sites need to remain independent and the pancreas’ biological response at the various doses need to be quantifiable given the proximity of pancreatic tissue to a particular blood vessel, within a maximum amount of time permitted to occlude the vessel. An anatomically correct pig pancreas model was generated from contrast-enhanced CT images to execute dose-escalation simulations by constructing an *in-silico* 3D pancreas with the major blood vessels in and around the pancreas shown in Fig. 12(a). The model comprises three layers of the pancreatic tissue (shown in orange), the arterial blood vessel (in red), and the vein (in blue) with assigned optical properties based on the literature for aortic tissues.^53^^,^^54^ The photon sources were modeled as cylinders with a 1 mm radius and 10 mm length. Figure 12(b) visualizes the resulting fluence around multiple sources placed simultaneously into the pancreas. The extent of the tissue destruction based on the photodynamic threshold model^55^^–^^57^ as a function of fluence can thus be utilized for experimental planning. Simulations also provide the maximum power permissible to prevent thermal damage to the vessel wall, by limiting the fluence rate at the intima to $<300\;\mathrm{mW}\;{\mathrm{cm}}^{-2}$. Figure 12(c) shows a cross-sectional line between two illumination points. The fluence along this line shown in Fig. 12(d) illustrating that the maximum permissible intima surface irradiation is not breached and that while there is an overlap of the fluence rate profiles it occurs only after a fluence rate attenuation exceeding three orders of magnitude equivalent to more than three effective attenuation coefficients. It is up to the experimental planning to ensure that this fluence overlap will not result in an overlap in tissue necrosis, to enable evaluation of each illumination spot separately.

## 6 Conclusion

In this work, we introduced FullMonteWeb, an open-source user-friendly web-based software with a graphical user interface for iPDT modeling and optimization. The tool can perform Monte Carlo simulations of light propagation in biological tissues, along with iPDT plan optimization. It is flexible in the source type and tumor location and size and provides in-browser visualization of both the problem geometry and results. FullMonteWeb leverages AWS to install and run the required libraries and software. We have shown that the best choice of AWS instance depends on the problem type and size and have provided some insight on what instance to choose based on a runtime-cost trade-off. Finally, we have highlighted how to leverage the software in different applications by showing how to extract the optical properties in a liver phantom and how to optimize the placement of light diffusers in a pig-pancreas iPDT experiment.

## 7 Appendix: FullMonteWeb Walkthrough

### 7.1 Treatment Plan Evaluation

To run FullMonteSW on FullMonteWeb, a series of input pages guide users intuitively to provide the necessary files and parameters for simulations. As shown previously in Fig. 5, the first page prompts the basic information, including the input mesh file, mesh units, types of energies to record, and more. In the back-end, FullMonteWeb takes all the selections and generates a Tcl script that runs FullMonteSW on the provided EC2 virtual machine.

**Fig. 13:**
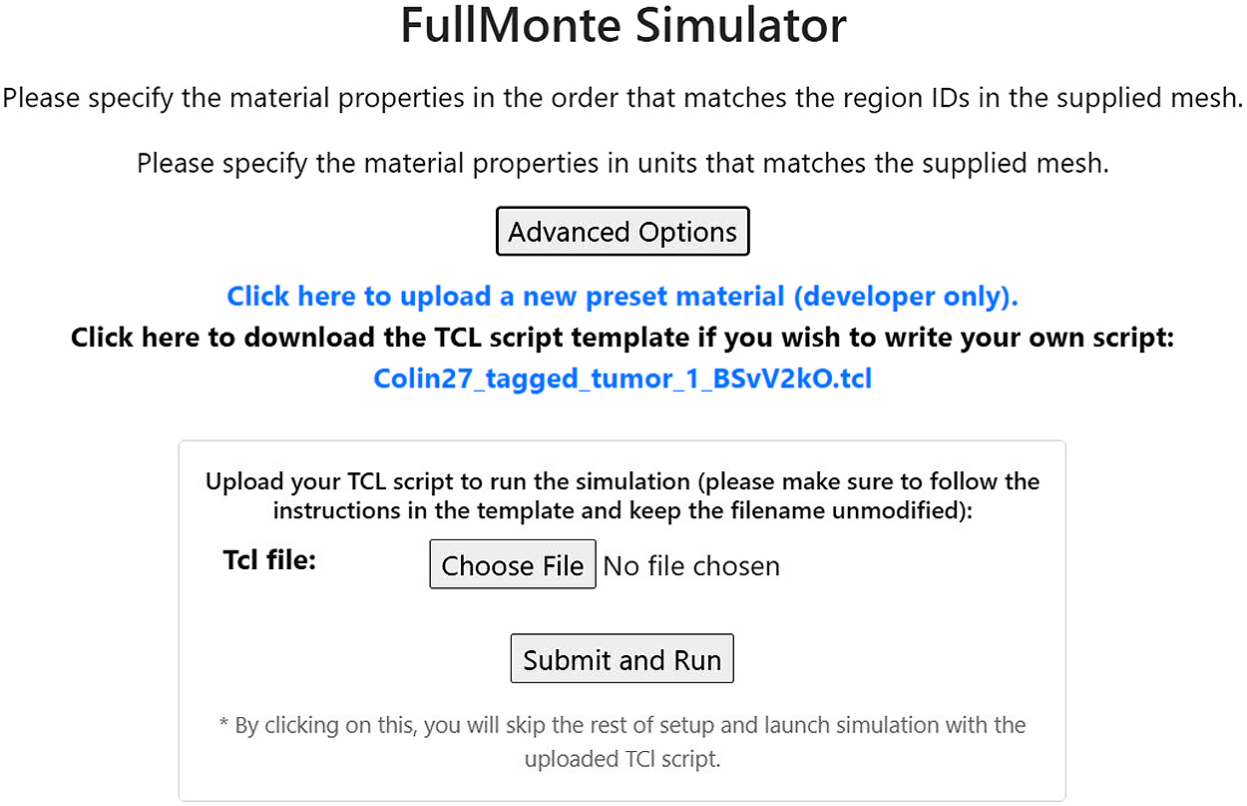
Advanced options for FullMonte simulation.

The second page prompts for the mesh’s region properties and allows users to save certain regions as presets for future quick selections. As shown in Fig. 13, advanced users can download the semifinished Tcl script from this page, manually complete the script, upload it back to FullMonteWeb, and skip the rest of the setup procedures.

The third page asks for the light source types and positions. The 3D visualization feature providing a visual model of the input mesh and its surrounding space aids users in selecting light source locations. The confirmation page provides a chance for users to review the settings and make any final changes to the Tcl script before launching the simulation. All Tcl scripts downloaded from these steps are prepared with comments to guide the user toward completing it.

**Fig. 14:**
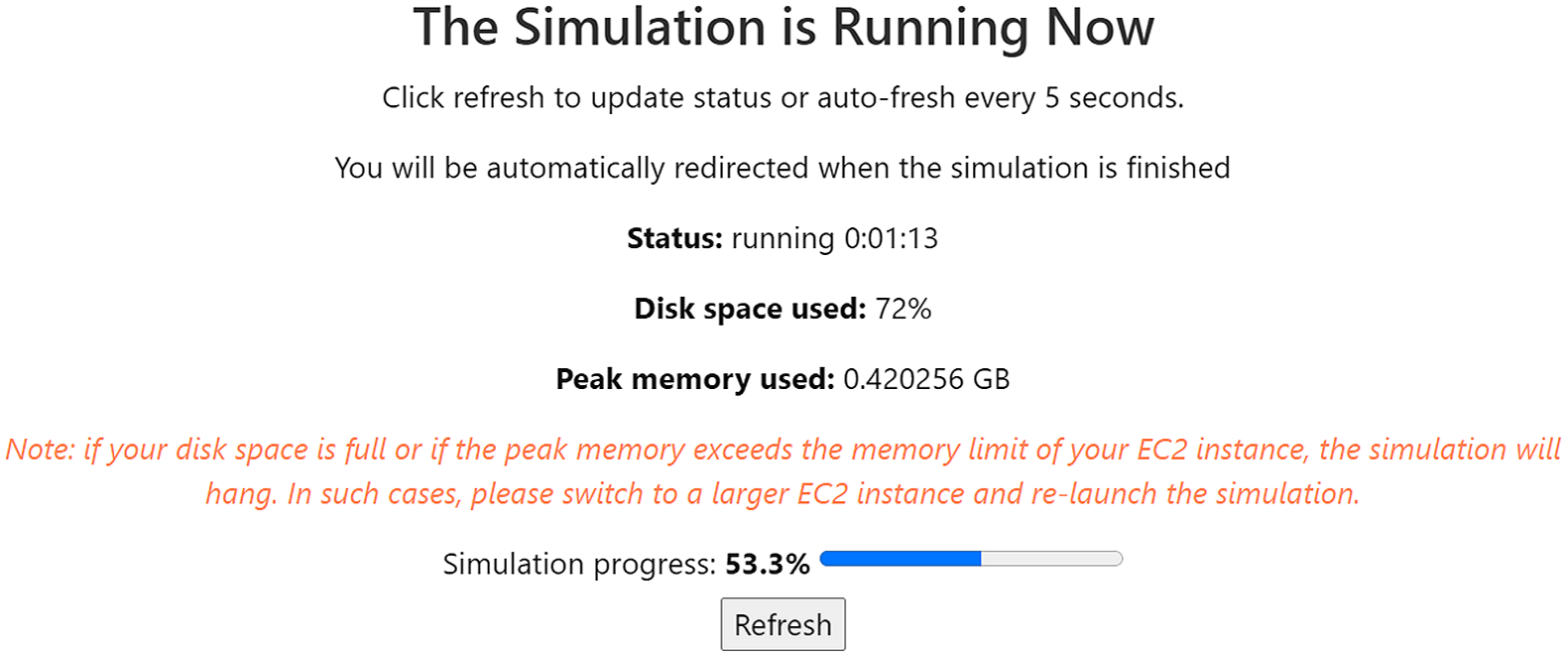
FullMonte simulation progress.

Upon confirmation, a progress page appears providing feedback on the execution progress in real time, as shown in Fig. 14. Upon completion, the simulation logs page is presented, providing potential warnings and errors that occurred during the simulation. Users can visualize the results, or download the Tcl script, input files, and output files via the simulation history page.

### 7.2 Treatment Plan Creation

To run PDT-SPACE, the user is guided by multiple input pages to provide the necessary files and parameters for software execution. A Mosek^58^ license needs to be obtained and uploaded to PDT-SPACE to access its Fusion C++ API library for solving the convex optimization problem. The license can be obtained by following the link provided on the upload page.

Next the user is asked to specify the input mesh, optical and issue property files, the number of photon packets, wavelength, and the tumor weight. The tumor weight is required when optimization is to be executed to maximize tumor destruction while minimizing OAR damage. Experienced users can specify the pruning normalization factor to scale the light dose thresholds. Subsequently, the user can select the source type, placement type, and upload an initial placement file containing this information. In the back-end, FullMonteWeb takes all the settings and generates a parameter script that contains all input parameter names and values for running PDT-SPACE on the specified EC2 instance.

**Fig. 15:**
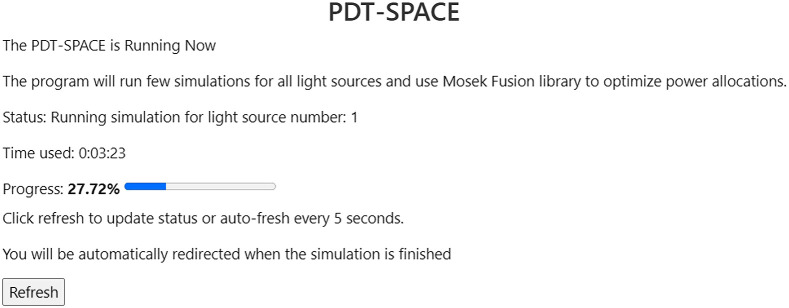
Progress page when running PDT-SPACE.

To launch PDT-SPACE optimization, FullMonteWeb executes two scripts that are created during AWS setup. The first script is to download and open the docker image, which contains the PDT-SPACE library on the user’s AWS account. The second script is to set the running environment and execute the PDT-SPACE command with the auto-generated parameter file. Once confirmed, users will be redirected to a progress page, as shown in Fig. 15.

**Fig. 16:**
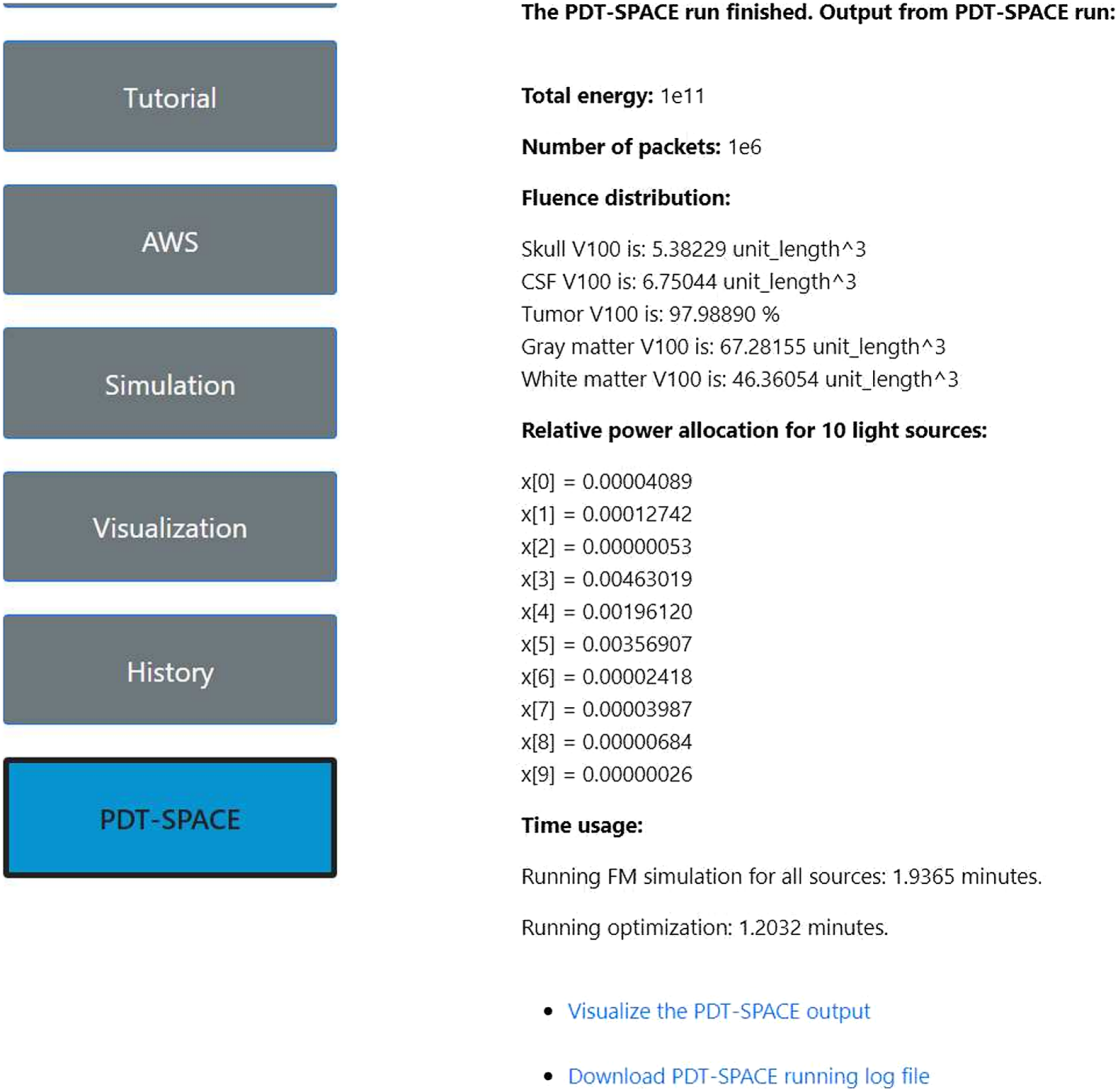
An example results page on FullMonteWeb after running PDT-SPACE.

Once the optimization completes, the website will automatically redirect users to a results page, as shown in Fig. 16. The page contains total energy, number of packets, fluence distribution across all material regions, relative power allocation for the specified light sources, and optimization run time. Users can then visualize the PDT-SPACE output or download the log files from the link at the bottom of the page.

### 7.3 Robustness and Security

**Fig. 17:**
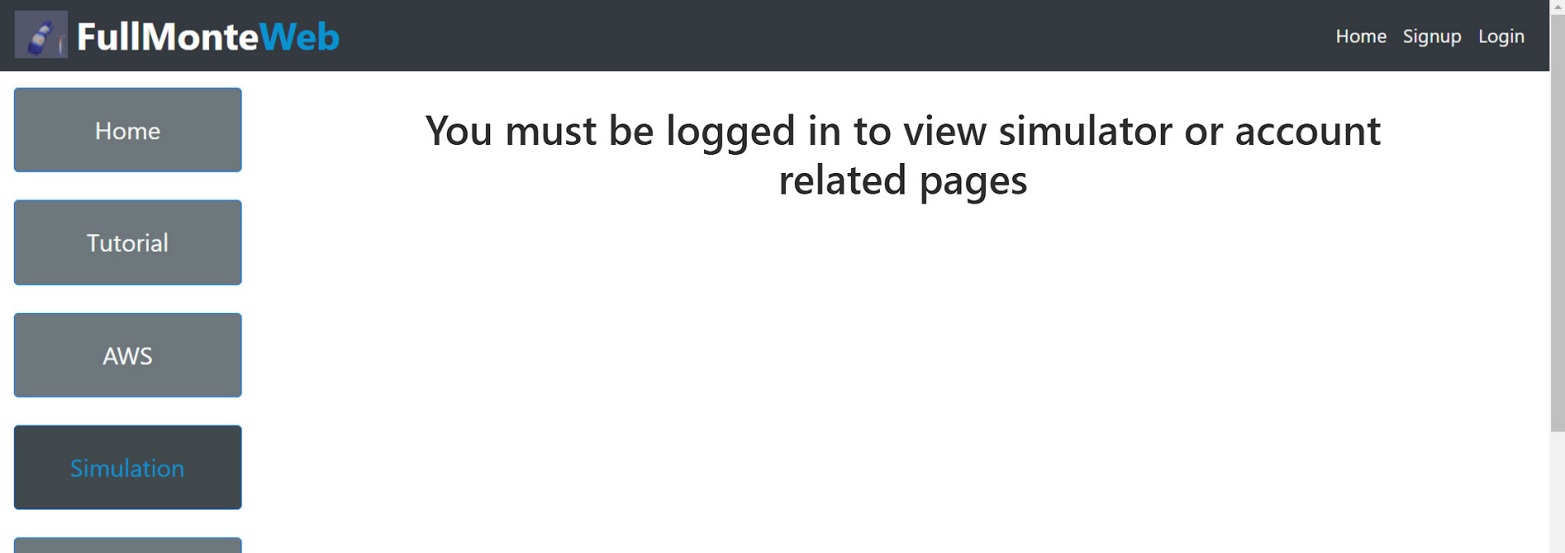
Error when user attempts to use core functions without logging in.

To ensure that users can only access their own files, all user data are protected by their accounts. As shown in Fig. 17, if users attempt to run FullMonteSW, PDT-SPACE, visualizers, or to access the simulation history page without logging in, they will not be able to proceed.

**Fig. 18:**
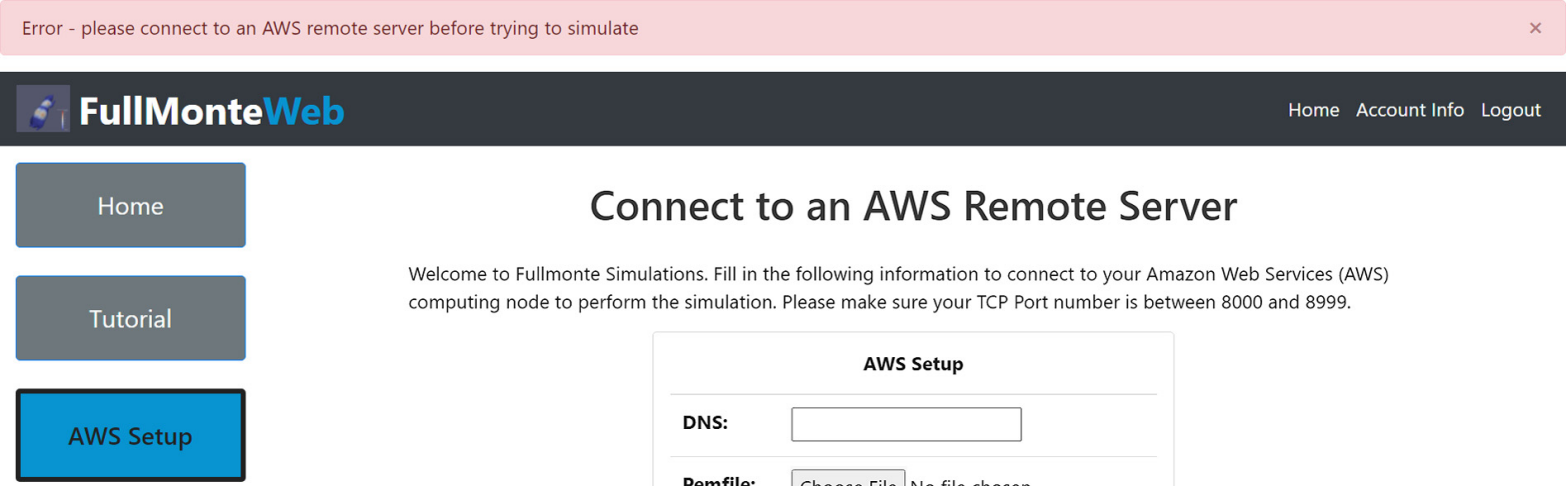
Error when user attempts to run simulation, optimization, or visualization without setting up a valid EC2 virtual machine.

Since FullMonteSW, PDT-SPACE, and visualizer computations cannot be done in the browser, users are required to set up an AWS virtual machine. If FullMonteWeb cannot detect a valid virtual machine setup on the account, the user will be redirected to the AWS Setup page, as shown in Fig. 18.

**Fig. 19:**
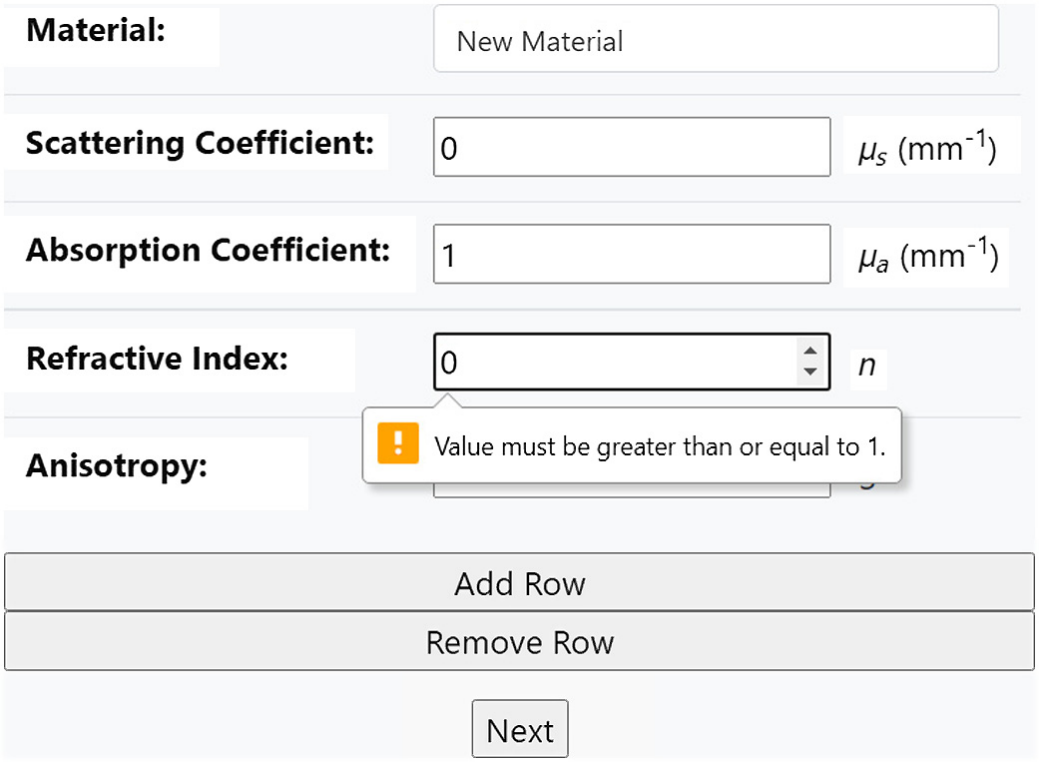
Error when user enters an invalid input.

To ensure the website’s reliability against invalid inputs and mismatches between mesh and specified material properties, there are several restrictions and validation modules implemented in the back-end. For example, the refractive index should be at least 1, so if the user specifies a value outside of this range, they will be alerted, as shown in Fig. 19. Furthermore, mesh files are typically specified in units of centimeters or millimeters, so users are required to specify the mesh unit when they upload the mesh file. All inputs following the upload should be in the same unit, so the website automatically converts all preset information to the same unit.

### 7.4 Visualization

#### 7.4.1 Dose-volume histogram

**Fig. 20:**
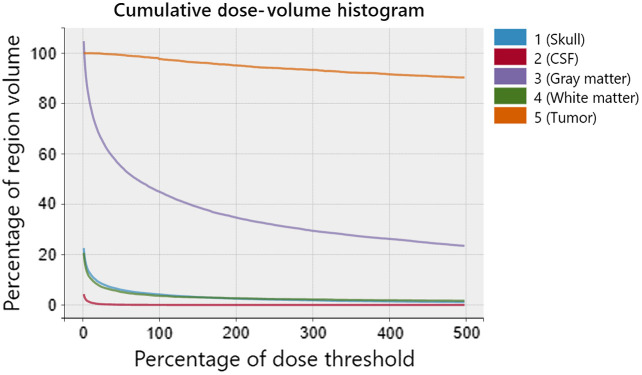
A dose volume histogram generated from PDT-SPACE output of an iPDT plan for a brain tumor with a $32\;{\mathrm{cm}}^{3}$ volume in the frontal lobe.

When the visualization feature is launched after a successful FullMonte simulation, the user will be prompted to upload a tissue properties file containing the threshold fluence for each region. FullMonteWeb will then automatically generate a DVH and display it on the web page. Likewise, a DVH will also be generated after a successful PDT-SPACE run but without the step of uploading a tissue properties file (see Fig. 20). A DVH serves to show the distribution of fluence (energy intensity) throughout each body tissue region. With this graph, the user can understand the volume of healthy tissue and tumor after receiving a certain percentage of its dose threshold. From the figure, the x axis represents the percentage of fluence dose with respect to the dose threshold for each tissue in the mesh and the y axis represents the percentage of a region that receives at least a given dose.

#### 7.4.2 3D interactive visualizer

As shown previously in Fig. 9, the user is able to interactively zoom in and out, as well as move and rotate the 3D model by dragging the model with the mouse. Moreover, with the paraview visualizer, the user can also add rendering filters to the 3D model, such as clip, slice, and threshold filters. With these filters, the user is able to see the inner property of meshes and have a better understanding of the treatment plan. For example, the user could first apply a threshold filter on the mesh and set the threshold range between regions 4 and 5, then apply a slice filter on the mesh so all other regions are rendered as a slice along the user-specified plane except for regions 4 and 5.

## Code, Data, and Materials Availability

All the FullMonte tools, including FullMonteWeb are available at Ref. 45. The source code of the tools and all used meshes in the manuscript are available at https://gitlab.com/FullMonte.
